# A case of refractory urethritis with repeated doctor shopping

**DOI:** 10.1002/iju5.12415

**Published:** 2022-01-22

**Authors:** Koki Maeda, Katsumi Shigemura, Yong‐Ming Yang, Yuzo Nakano, Soichi Arakawa, Masato Fujisawa

**Affiliations:** ^1^ Department of Urology Kobe University Graduate School of Medicine Kobe Japan; ^2^ Department of Urology Sanda City Hospital Sanda Japan

**Keywords:** antibiotic resistance, doctor shopping, sexually transmitted disease

## Abstract

**Introduction:**

In the field of sexually transmitted diseases, resistance and diversification of causative organisms are becoming a problem. We report a case in which the course of the disease was complicated by doctor shopping.

**Case presentation:**

A man in his 40s visited his local doctor for painful urination and cloudy urine. Due to the lack of improvement in symptoms after antibiotic treatment, he self‐selected to visit six hospitals in just five months. He visited our clinic only a few times and then stopped coming.

**Conclusion:**

Doctor shopping, as well as self‐diagnosis and self‐treatment, will continue to increase. Patient education is important, but medical professionals also need to be aware of the possibility of doctor shopping when treating patients.

**Abbreviations & Acronyms**
AZM: azithromycin
DOXY: doxycycline
ESBL: extended‐spectrum β‐lactamases
LVFX: levofloxacin
*M. genitalium*
: *Mycoplasma genitalium*
MOFX: moxifloxacin
STFX: sitafloxacin

**Keynote message**

We report here a case of refractory urethritis with repeated doctor shopping. We need to be aware of the possibility of doctor shopping when treating patients.

## Introduction

The emergence of multidrug‐resistant bacteria is a problem in the field of sexually transmitted diseases as well as in urinary tract infections. Neisseria gonorrhoeae is a particularly serious problem^1^, ^2^, ^3^, ^4^ and non‐gonococcal non‐chlamydial urethritis, such as mycoplasma, is said to account for 30% of cases.^5^

The family doctor system is not very popular in Japan and doctor shopping, where patients visit multiple medical institutions at the same time for the same disease, is a common problem. We report a case of difficulty in treating a sexually transmitted disease due to poor compliance with the examination, testing, and medication.

## Case presentation

**Fig. 1:**
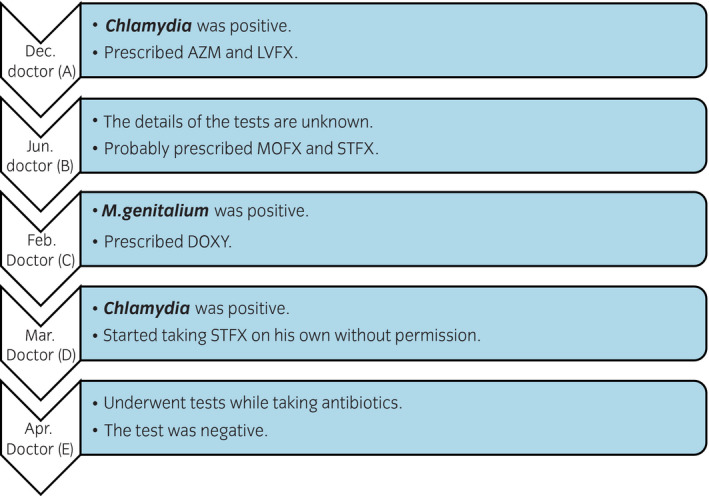
Summary of treatment progress. The patient had been to several medical institutions in a short period of time. While seeing Dr. (D), the patient had interrupted and restarted antibiotics prescribed by another medical institution at his own discretion.

A man in his 40s visited his local physician (A) with a chief complaint of pain during urination and cloudy urine in December. Figure 1 shows a summary of the course of treatment. The local doctor (A) suspected urethritis due to a sexually transmitted infection and prescribed a single dose of AZM and 5 days of LVFX. The PCR test results were positive for *chlamydia* and negative for *gonorrhea*. Antibiotic treatments improved the pain during urination, but the cloudy urine persisted.

In January, the patient visited a hospital (B) in Hawaii due to persistent cloudy urine. The details of the tests and treatment are unknown. It seems he was treated with several oral and intravenous antibiotics such as MOFX and STFX. In February, the test was negative for causative bacteria. However, he felt distrustful and dissatisfied because he continued to feel discomfort during urination similar to prostatitis and test results showed he had ESBL*‐producing E. coli*. He stopped visiting the hospital (B) at his own discretion.

The patient came back to Japan and went to see another local doctor (C) in March. Once again, various culture and bacteriological tests were submitted, and *Mycoplasma genitalium* was positive. Based on the susceptibility results, oral treatment with DOXY for 10 days was administered. However, the pain during urination and cloudy urine flared up even with the treatment, so he stopped seeing his local doctor (C) on his own judgment and went to see another hospital (D) in March. The test results were positive for chlamydia and negative for mycoplasma, but he had been taking STFX internally at his own discretion even before the test results came back.

In addition, he visited our university hospital (E) at the end of March for a flare‐up of symptoms. The PCR test for gonorrhea and chlamydia was negative, and urine culture was also negative. This test was done with the patient taking antibiotics internally at his own discretion. Then, without any further contact, his visits to our hospital ceased.

## Discussion

In this report, we examined a patient who moved from one medical institution to another over a short period of time due to recurrent urethritis, and who had stopped, continued, or even added to the antibiotics prescribed by each institution at his own discretion. Although this level of doctor shopping is rare, it is common to see doctor shopping in Japan. A number of causes have been cited such as comorbidities, poor patient satisfaction, and active substance abuse,^6^ but Japan's universal health care system is probably one of the causes since nearly everyone is covered by national health insurance, and covered patients pay only 20 ~ 30% of the medical fee.^7^

While patients are allowed to freely choose their medical institutions, doctor shopping has a major disadvantage. Duplicate testing at different medical institutions wastes medical costs and time and, depending on the test, may even pose a health risk to the patient.^6^ In addition, unintentional concomitant use of therapeutic agents may reduce their efficacy and increase side effects due to interactions,^8^ and especially during treatment for infectious diseases abuse or misuse of antibiotics may lead to the development of antibiotic resistance.^9^

In the field of urology, chronic prostatitis, overactive bladder, and interstitial cystitis tend to lead to doctor shopping because they do not improve easily with a long‐term course, but they are rarely reported. Doctor shopping by overactive bladder patients was reported in an article from Hong Kong^10^ including some of the causes and backgrounds of doctor shopping. In our case, the unpleasant experience of treatment failure is applicable.

In sexually transmitted diseases, resistance and diversification of causative organisms are becoming a problem, and as a result, empirical antibiotic therapy is increasingly leading to initial treatment failure.^11^ In addition, the use of antibiotics prescribed from multiple medical institutions may make it difficult to interpret culture test results.

Doctor Shoppers also tend to be younger and better informed about medical specialties.^12^ The widespread use of the Internet has made it easier for people to search for their own symptoms and self‐diagnose themselves.^13^ Sexually transmitted diseases are also common among young people, and symptoms are typical and similar, making self‐diagnosis easy. If the possibility of treatment failure due to resistant bacteria is not understood, the relationship between doctor and patient may be easily lost when the treatment fails. As a result, treatment failure leads to doctor shopping.

Self‐diagnosis, self‐judgmental treatment discontinuation, and doctor shopping will not disappear in the future. The national policy of linking drug and treatment histories to health insurance cards may help reduce doctor shopping, but it will require huge costs.^14^ Also, if all medical institutions do not subscribe, the doctor‐shoppers will just keep shopping, no matter how far away they are.^15^

Medical professionals need to take into account the possibility of doctor shopping and conduct basic medical interviews, explain the possibility of treatment failure, check medications, and work more closely with other medical professionals.

## Author Contributions

**Koki Maeda:** Conceptualization; Data curation; Formal analysis; Investigation; Methodology; Resources; Software; Writing – original draft; Writing – review & editing. **Katsumi Shigemura:** Validation; Visualization; Writing – original draft; Writing – review & editing. **Yong‐Ming Yang:** Data curation; Formal analysis; Resources; Software. **Yuzo Nakano:** Supervision; Validation. **Soichi Arakawa:** Project administration; Supervision. **Masato Fujisawa:** Project administration; Supervision.

## Conflict of interest

Not applicable.

## Approval of the research protocol by an institutional reviewer board

Not applicable.

## Informed consent

Not applicable.

## Registry and the registration no. of the study/trial

Not applicable.
